# Impact of lipid asymmetry on membrane biophysical properties: Insights from molecular dynamics simulations

**DOI:** 10.1002/qub2.89

**Published:** 2025-01-27

**Authors:** Yong Zhang, Jizhong Lou

**Affiliations:** ^1^ Key Laboratory of Epigenetic Regulation and Intervention Institute of Biophysics Chinese Academy of Sciences Beijing China; ^2^ University of Chinese Academy of Sciences Beijing China

**Keywords:** cholesterol distribution, membrane asymmetry, membrane electrostatic potential, membrane pressure, molecular dynamics simulation

## Abstract

Asymmetry between outer and inner leaflets of cell membrane, such as variations in phospholipid composition, cholesterol (CHOL) distribution, stress levels, and ion environments, could significantly influence the biophysical properties of membranes, including the lateral organization of lipids and the formation of membrane nanodomains. To elucidate the effects of lipid component, lipid number mismatch, CHOL concentration asymmetry, and ionic conditions on membrane properties, we constructed several sets of all‐atom, multi‐component lipid bilayer models. Using molecular dynamics (MD) simulations, we investigated how membrane asymmetry modulates its biological characteristics. Our results indicate that CHOL concentration, whether symmetric or asymmetric between the leaflets, is the primary factor affecting membrane thickness, order parameters of the lipid tail, tilting angles of lipid molecules, water permeability, lateral pressure profiles, and transmembrane potential. Both low and high CHOL concentrations significantly alter lipid bilayer properties. Inducing cross‐leaflet stress by mismatching lipid numbers can modify lipid order parameters and the tilting angles but has only mild effect on lateral pressure profiles and membrane thickness. Additionally, we found that transmembrane potential, generated by ions concentration differences across the membrane, can influence water permeability. Our findings expand the current understanding of lipid membrane properties and underscore the importance of considering CHOL and phospholipid asymmetry in membrane biophysics. The membrane models developed in our study also provide more physiological conditions for studying membrane proteins using MD simulations.

## 1 INTRODUCTION

Cellular membranes play an irreplaceable role in separating the contents of cells from their surroundings and regulating communications among cells, as well as between cells and extracellular environment. The lipid nano‐environment is also thought to regulate membrane protein structure and function [1]. A fundamental understanding of the structure of cellular membranes is required to uncover their functions. The dynamical lipid bilayer, as suggested by fluid mosaic model, has been adopted widely to depict cellular membranes. Extensive studies have confirmed that the components in the two leaflets of lipid membranes are asymmetric [2, 3, 4]. Asymmetry is an inherent property of most biological membrane, resulting from the differential distribution of phospholipids and cholesterol (CHOL). For example, in the plasma membrane of most animal cells, the outer leaflet mainly consists of phosphatidylcholine (PC) and sphingomyelin (SM), whereas the inner leaflet primarily comprises phosphatidylethanolamine (PE), PC, phosphatidylserine (PS), and a small number of various phosphatidylinositol species [5, 6, 7]. CHOL is abundant in both leaflets of the plasma membrane at a molar ratio ranging from about 10% to 50% [8]. Although some debate remains, recent studies have shown that CHOL concentration in the outer leaflet is evidently higher than in the inner leaflet, indicating an asymmetric distribution of CHOL in cell membranes [9, 10, 11].

Component asymmetry inevitably leads to more complex membrane properties. Various phospholipids, characterized by charged or uncharged headgroups, and saturated or unsaturated aliphatic tails of different length, exhibit diverse physical and chemical features, such as the area per lipid, which indicates the average surface area occupied by a single lipid molecule [12, 13, 14]. Due to the presence of different components with varying areas per lipid in the two leaflets, the number of lipids in the two leaflets also differs, as observed in several studies [15, 16]. The role of CHOL and its modified derivatives is indispensable and has been extensively studied using experimental and computational methods [17, 18, 19, 20, 21]. CHOL significantly affects the permeability, fluidity, and mechanical properties of the membrane [22, 23]. Its ordering and condensing effects on the membrane have also been noted. CHOL preferentially associates with saturated lipids, which can induce phase separation between ordered and disordered lipid nanodomains [24]. Thus, the extensive diversity of phospholipids and the variable CHOL abundance in the two leaflets promote membrane complexity and endow it with adjustable properties.

Molecular dynamics (MD) simulation has been proven to be an effective method to study membrane properties, including asymmetry, over the past several decades. It has shown that area asymmetry of lipid bilayers is responsible for the fusion of viruses with target cells, mediated by insertion of fusion peptide into the membrane [25]; the effect of asymmetric lipid distribution on the electrostatic potential across the membrane has been indicated [26, 27]; changes in bilayer properties upon replacing uncharged PC lipids with charged lipids in one monolayer of the membrane have been investigated [28]; and the stability of symmetric and asymmetric membranes has been quantified [29, 30]. However, in these studies, the lipid bilayer models were built without considering the possible area difference between the outer and inner leaflets. In one study, Sachs et al. considered the area difference of leaflets when studying the interleaflet interaction and asymmetry of phase‐separated lipid bilayer using coarse‐grained MD method, but only the PC lipids were used in their model [31]. An asymmetric multi‐component membrane model that considered leaflet area matching but excluded CHOL was used to study water permeation across the membrane [32]. More recently, many computational modeling has focused on approximating realistic cell membranes to study their global properties [33, 34, 35]. The influence of individual lipids was hard to elucidate due to the presence of many lipid components.

Few studies involving multiple lipid components consider both phospholipid and CHOL asymmetry at the atomistic level. In this study, we present extensive atomistic simulations to quantitatively decipher the important roles of lipid asymmetry on membrane properties by considering the component differences of lipid molecules between leaflets, the match/mismatch of lateral area caused by lipid asymmetry, the phospholipid/CHOL ratio, and the ion environment differences on the outer and inner sides of the membrane. We constructed six distinct sets of all‐atom, multi‐component lipid bilayer models, systematically manipulating CHOL concentration, lipid counts, and ion concentrations (Table S1). We first constructed models with matched total areas (asymPL) between the two leaflets based on the simulated lateral area of the symmetric membrane models (INNER and OUTER), and investigate how CHOL modulates the structure and lateral pressure profiles of the asymmetric lipid bilayer in the equilibrium state. Then, lipid bilayer models with area mismatches (STRESS) were induced by reducing the lipid number of one leaflet to uncover the influence of intrinsic stress on membrane properties. Subsequently, lipid bilayer models with differed phospholipid/CHOL ratios in the two leaflets (asymCHOL) were constructed to reveal the role of CHOL asymmetry. Furthermore, to validate the influence of ion environment and membrane potential, we crafted lipid bilayer models by strategically positioning two lipid bilayers against each other in one system (PHYSIOL). The results of this study provide quantitative information on the biophysical and biochemical properties of membrane lipids, and the membrane models generated herein will benefit future membrane protein studies using MD simulations.

## 2 RESULTS

### 2.1 Cholesterol modulates the lateral area and geometry of asymmetric lipid bilayer

Lipid asymmetry, coupled with varying phospholipid/CHOL ratios, results in different molecular counts within a specific area, influencing membrane geometries. Given CHOL’s pivotal role in regulating the stability, the stiffness, and the order of the membrane, we first investigate its influence by constructing symmetric and asymmetric lipid bilayer models with equal phospholipid/CHOL ratio between two leaflets.

**FIGURE 1:**
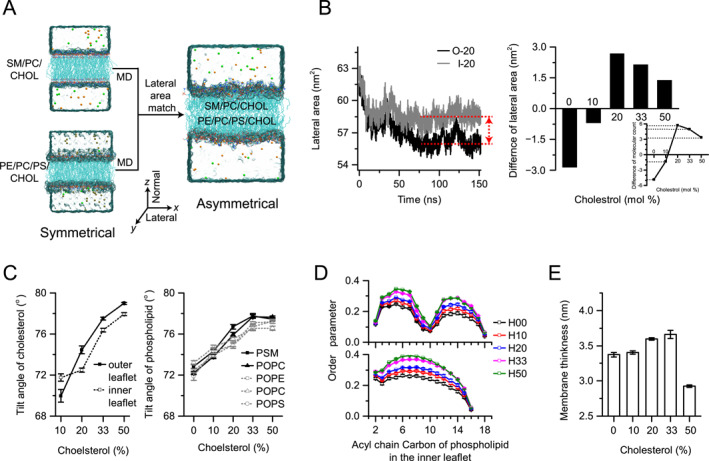
Cholesterol (CHOL) modulates the lateral area and geometries of lipid bilayer. (A) Schematics of constructing the asymmetrical lipid bilayer models (asymPL) by adjusting the number of lipids in outer and inner leaflets to match the lateral area determined from the simulations of the symmetrical lipid bilayer models (OUTER and INNER). (B) Representative time‐courses of the change in the lateral area of the symmetrical lipid bilayers (20% CHOL, left). The difference of lateral area between corresponding OUTER and INNER models was compensated by adjusting the number of lipids in the OUTER or INNER models. The difference in lateral area between the OUTER model and the corresponding INNER model was calculated (right). The number of each kind of lipid was computed based on the lateral area differences (inset). (C) The average tilting angles of CHOL (left panel) and phospholipids (right panel) in five asymPL models. Results for the outer leaflet are shown in solid black lines, and those for the inner leaflet are shown in dotted gray lines. (D) Order parameters for unsaturated (top) and saturated (bottom) tails of phospholipids in the inner leaflet in the asymPL models. (E) The membrane thickness of asymPL models, defined by the distance between the maximal mass densities of two leaflets along *z* axis. Error bars in (C–D) are SEMs of two independent simulations.

Initially, two sets of symmetric lipid bilayer models were built using typical phospholipid components observed in the outer and inner leaflets of cellular plasma membrane. These are, the outer leaflet models (OUTER), consisting of SM and PC lipids and the inner leaflet models (INNER), consisting of PE, PC, and PS lipids. By altering the phospholipid/CHOL ratio, five models were built in each set (Figure 1A and Table S1). The changes in the lateral areas of the symmetric lipid bilayer models during the simulations are monitored (Figure 1B left and Figure S1). It was found that the lateral areas of both OUTER and INNER models decreased monotonically with increasing CHOL concentration, dropping from about 70.7 nm^2^ to 49.5 nm^2^ for OUTER models and from about 67.8 nm^2^ to 50.9 nm^2^ for INNER models (Figure S1A). This reduction can be attributed to the smaller area per lipid occupied by CHOL compared to phospholipids, typically about 0.3 nm^2^ for CHOL and about 0.6 nm^2^ for phospholipids [36]. Moreover, the condensing effect of CHOL further decreases the area per lipid [37]. Interestingly, the fluctuation of lateral area during the simulation, quantified by the standard deviation of area changes, reduces with increasing CHOL concentrations, implying the stabilizing and condensing effect of CHOL on lipid bilayer structure (Figure S1A).

Previous studies have shown that the optimal choice to construct an asymmetric lipid bilayer model is to match the lateral surface area of the two leaflets [16]. Using a similar strategy, a set of five asymmetric lipid bilayer models (asymPL) were constructed with CHOL concentrations ranging from 0% to 50%. By comparing the lateral area differences between OUTER and INNER models with the same CHOL concentration, it is found that lateral area of OUTER models is about 2.9 nm^2^ and 0.9 nm^2^ larger than that of INNER models for 0% and 10% CHOL, and about 2.7 nm^2^, 2.0 nm^2^, and 1.5 nm^2^ smaller than that of INNER models for 20%, 33%, and 50% CHOL (Figure 1B right). The behavior might result from the association of CHOL with the saturated SM fatty acid chain, which tends to promote the membrane ordering thus reduces that lateral area. To match the lateral area of outer and inner leaflets, the offsets of lipid count between the outer and inner leaflets are calculated based on the difference of average area while trying to maintain the ratio of lipid components (Figure 1B insert and Table S1). The asymPL models were then constructed (Figure 1A and Table S1), whereupon MD simulations were conducted. The tilting angle of lipids, thickness of lipid bilayer, and order parameter of lipid tails were measured for these models.

The tilting angle of a lipid describes the molecule’s orientation relative to the lipid bilayer plane. The most probable tilting angle of CHOL increased from about 71° to 78° monotonically with rising CHOL concentration in four asymPL models with CHOL (Figure 1C, left), similar to that observed in lipid bilayers containing only PC phospholipid and CHOL [37]. The most probable tilting angle of each phospholipid component increased from about 73° to 77° when CHOL concentration increased from 0% to 33%, and decreased slightly at 50% CHOL concentration (Figure 1C, right). These results imply that both phospholipids and CHOL become closer to perpendicular to the bilayer plane with increasing CHOL concentration. Considering the differences between the two leaflets, it is found that the tilting angle of CHOL was larger in the leaflet with more lipid molecules.

The degree of membrane order is quantified by the order parameter of the lipid tails. The order parameter profiles of asymPL models showed that the ordering of both saturated and unsaturated lipid tails increased with increasing CHOL concentration (Figure 1D), consistent with other studies and reinforcing the importance of CHOL concentration in shaping membrane properties and function [37, 40, 41].

The membrane thickness, defined as the distance between two peaks in the mass distribution curves of lipid molecule (Figure S1B), exhibited an initial increase with the CHOL concentration up to about 33%, followed by a marked decrease for 50% CHOL concentration (Figure 1E). This trend correlated well with the changes in tilting angles and order parameters of phospholipids except at 50% CHOL concentration (Figure 1C, right). It is thought that the combined influence of phospholipids and CHOL molecules shapes the bilayer’s mass distribution. Phospholipid molecules, being larger and elongated, contrast with the smaller, shorter CHOL molecules that primarily reside in the hydrophobic domain of the phospholipids. Consequently, their respective mass density profiles do not coincide along the bilayer’s *z*‐axis, with CHOL’s profile positioned closer to the bilayer’s center (Figure S1C). Accordingly, though the phospholipid’s thickness increases with rising CHOL concentration (Figure S1D), the membrane thickness is reduced when CHOL concentration reaches 50%. That is, optimal CHOL concentration could maximize the membrane thickness. Previous atomic force microscope and X‐ray diffraction studies showed that the difference in membrane thickness between raft and nonraft membrane domains reaches about 0.6–0.9 nm [38, 39]. Together with our results, it is suggested that the CHOL concentration should be higher in raft membrane domain than that in the nonraft membrane domain, in agreement with the common belief that the membrane raft is formed by compact packing of the CHOL with SM lipids.

### 2.2 Effects of CHOL asymmetry

**FIGURE 2:**
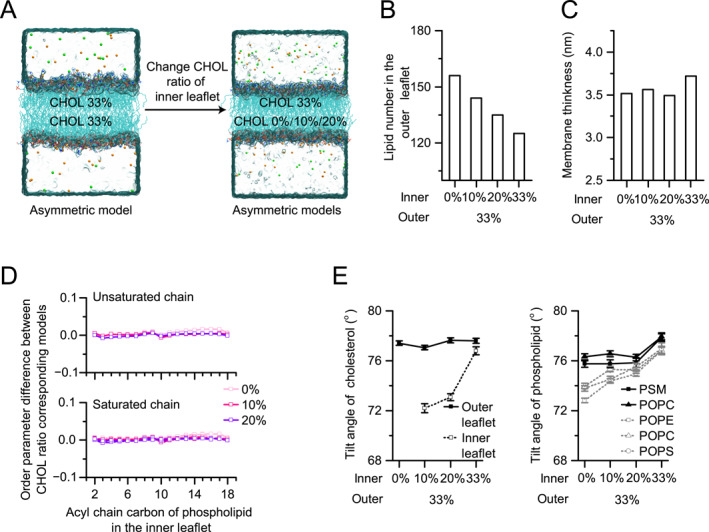
Effects of cholesterol (CHOL) asymmetry. (A) Schematics of CHOL asymmetry model (asymCHOL) construction. The O33 model from OUTER set was used as the outer leaflet, the I00, I10, or I20 models from INNER set were used as the inner leaflet. The models were combined by adjusting the lipid number in the OUTER models to match the lateral area of the two leaflets. (B) Total number of lipids in the outer leaflet used in the models. The lipid number of the inner leaflet remains unchanged, the lipid number in the outer leaflet was changed to match the lateral area of the two leaflets. (C) Reducing CHOL concentration in the inner leaflet reduces the membrane thickness of the bilayer. (D) The differences in the order parameter between the asymCHOL models and their corresponding asymPL models (H00, H10, and H20) for unsaturated (top) and saturated (down) tails in the inner leaflet. (E) The average tilting angles of CHOL (left) and phospholipids (right) in asymPL H33 model and the asymCHOL models. The results for the outer leaflet are shown in solid black lines and these for the inner leaflet are shown in dotted gray lines. Error bars are SEMs from one simulation trajectory.

Biological membranes are highly dynamic, and recent studies suggest uneven CHOL distribution across the two leaflets, with higher concentrations in the outer leaflet [9, 10]. Moreover, the formation of lipid rafts through the coupling of CHOL and SM within the outer leaflet may further increase CHOL asymmetry between the leaflets in certain regions [42]. To address the impact of CHOL asymmetry, we constructed a set of three asymmetric lipid bilayer models (asymCHOL), each featuring distinct CHOL concentrations between the outer and inner leaflets. These models were crafted by aligning their lateral area with those simulated from OUTER and INNER models (Figure 2A–B and Table S1).

The simulation results of the asymCHOL models showed that membrane thickness decreased weakly (about 0.1–0.2 nm) along with the decrease of CHOL concentration in the inner leaflet (Figure 2C), whereas the density distribution became asymmetric in the normal direction of lipid bilayer (Figure S2A). The order parameters of lipid tails in both leaflets showed no significant difference from those in the corresponding leaflet of the asymPL models (Figure 2D and Figure S2B). This indicates that the order of lipid tails in a given leaflet is determined by the CHOL concentration within that leaflet, and is not affected by the CHOL concentration in the opposing leaflet. The tilting angles of both CHOL and phospholipids decreased with the reduction of CHOL concentration in the inner leaflet, consistent with those observed in the asymPL models (Figure 2E).

CHOL exhibits the capability to undergo free flip‐flop motions, as well as being facilitated by energy‐driven flippase or floppase, thereby modifying the membrane properties and affecting the function of membrane proteins [9]. Our simulations reveal that the CHOL concentration in each leaflet influences the structural properties of that leaflet but has only a mild direct effect on the opposite leaflet.

### 2.3 Effects of lipid number mismatch

**FIGURE 3:**
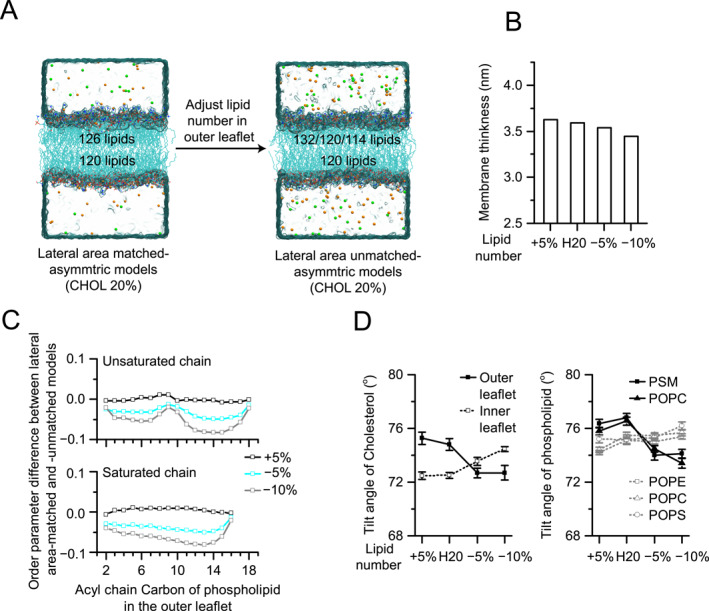
Effect of lipid number mismatch between the two leaflets. (A) Schematics of constructing the STRESS models. The number of lipids was altered in the outer leaflet of the asymPL H20 model, whereas the number of lipids in the inner leaflet remains unchanged. Three models were constructed: 5% increase of lipid number (+5%), 5% decrease of lipid number (−5%); and 10% decrease of the lipid number (−10%). (B) Changes in the membrane thickness of the STRESS models compared with that of the corresponding asymPL H20 model. (C) Order parameter differences between the STRESS models and asymPL H20 model for unsaturated (top) and saturated (bottom) lipid tails in the outer leaflet. (D) Comparison of the average tilting angles of cholesterol (CHOL) (left) and phospholipids (right) between the STRESS models and the asymPL H20 model. Results for the outer leaflet are shown in solid black lines and those for the inner leaflet are shown in dotted gray lines. Error bars are SEMs from one simulation trajectory.

Under certain circumstance, such as vesicle fusion or enhanced flippase/floppase function, the number of lipids in the two leaflets might shift away from equilibrium, inducing intrinsic stress between the leaflets. To reveal how lipid number mismatch may alter bilayer properties, we constructed a set of three asymmetric bilayer models (STRESS), introducing a mismatch in lipid numbers between the outer and inner leaflets by adjusting the lipid count in the outer leaflet, based on the asymPL H20 model (Figure 3A and Table S1).

The membrane thickness for all three STRESS models decreased slightly with the reduction in lipid number in the outer leaflet (Figure 3B), while the density distribution exhibited nonmonotonically changes with lipid number mismatch (Figure S3A). It is worth noting that in the outer leaflet, a 5% increase in lipid molecules slightly increased the order parameter of lipid tails, whereas a 5% or 10% decrease in lipid molecules diminished it dramatically (Figure 3C), likely due to the loosening of lipid tail packing. Intriguingly, the order parameters of lipid tail in the inner leaflet changed only moderately despite the mismatch (Figure S3B). The tilting angles of both CHOL and phospholipids decreased by 2°–3° compared to the asymPL H20 model, whereas those in the inner leaflet increased by 1°–2° (Figure 3D).

In cells, lipid number mismatch between the two leaflets may occur transiently and within limited regions, exerting minimal influence on the overall membrane environment. Our results are in coincident with a previous study on systemic lipid bilayer model comprising only single component [15].

### 2.4 Lipid asymmetry modulates lateral pressure profiles of membrane

Pressure inhomogeneity within membranes has been proposed to relate to the structure and function of specific membrane proteins, potentially activating them through changes in membrane pressure distribution [43, 44]. The lateral pressure across the normal direction of the lipid bilayer derives from local forces within the bilayer and is far from homogeneous. This heterogeneity arises from the disparate contributions of water, CHOL, and head groups and acyl chains of phospholipids to the overall pressure. Generally, a lateral pressure profile for a typical lipid bilayer without CHOL can be characterized by three distinct regimes: firstly, a repulsive contribution arising from electrostatic and steric interactions, as well as hydration repulsion between hydrophilic head groups; secondly, an attractive contribution attributed to the interfacial energy between water and the ester group of phospholipids, which tends to squeeze them together to minimize surface area; and thirdly, a repulsive contribution resulting from the steric and entropic interaction between acyl chains of phospholipids [8, 45].

**FIGURE 4:**
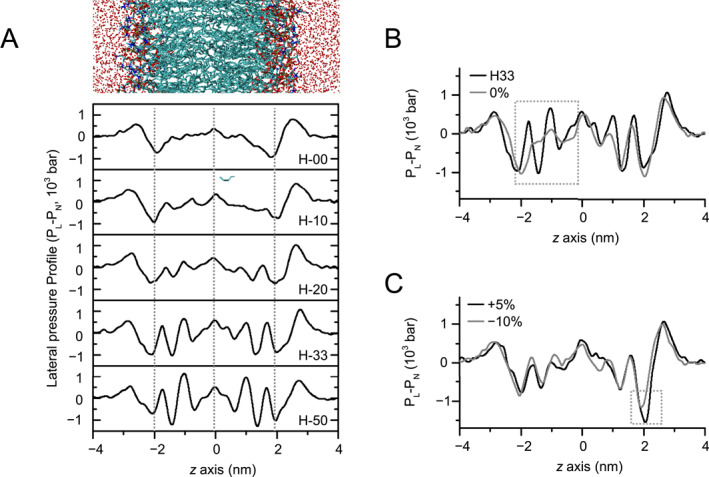
Lipid asymmetry modulates lateral pressure profiles of the membrane. (A) Lateral pressure profiles calculated from the simulations of the asymPL models. The lateral pressure profiles can be divided into different structural regions as indicated by the dotted lines. (B) Comparison of lateral pressure profiles for 0% asymCHOL model with the asymPL H33 model. The region with significant differences is marked with dotted box. (C) The lateral pressure profiles for the −10% and +5% STRESS models. The differing region is marked with a dotted box.

The lateral pressure profile for our asymPL H00 model aligns with the established rule (Figure 4A) and agrees with other studies on lipid bilayers without CHOL [46, 47, 48]. As CHOL concentration increases, the positive peak (2.5 nm < |*z|* < 3 nm) and the negative peak (1.8 nm < |*z*| < 2.3 nm) in the pressure profile do not change significantly. In contrast, the pressure profile within the hydrophobic region of the lipid bilayer alters dramatically, accompanied by pronounced fluctuations along the *z* axis (Figure 4A). The effect of CHOL on the lateral pressure in the hydrophobic region of the bilayer has been discussed previously, it is suggested that the pressure within the bilayer may change qualitatively for about 20% or higher CHOL concentrations in dipalmitoyl‐phosphatidylcholine‐cholesterol (DPPC‐CHOL) symmetric lipid bilayers [49]. Here we observed distinct alterations in the pressure profiles as CHOL concentration increases for membrane models with diverse phospholipids.

Due to the different lipid composition in the inner and outer leaflets, the pressure profiles also display asymmetric features. It is worth noting that the positive peak from hydrophilic head groups in the outer leaflet is nearly twice as large as that of the inner leaflet for most asymPL models. This disparity arises from the larger repulsive force exerted by the choline head group in the outer leaflet compared to the serine or ethanolamine head group in the inner leaflet (Figure 4A). In contrast, the negative peak resulting from the interaction of water and the ester group remains largely consistent between the outer and inner leaflets, indicating that this interaction is not affected significantly by the lipid head groups.

CHOL asymmetry further modulates the lateral pressure profiles. In the asymCHOL models, the lateral pressure profiles in outer leaflet are similar with that in the asymPL H33 model, while the in inner leaflet’s profiles resemble that of the asymPL model with the corresponding CHOL concentration (Figure 4B). Unexpectedly, in STRESS model with lipid number mismatch, the press profile remains largely unchanged, except for a modest variation in the lipid head region in the outer leaflet (Figure 4C).

### 2.5 Lipid components and ions concentration affect transmembrane electrostatic potentials

**FIGURE 5:**
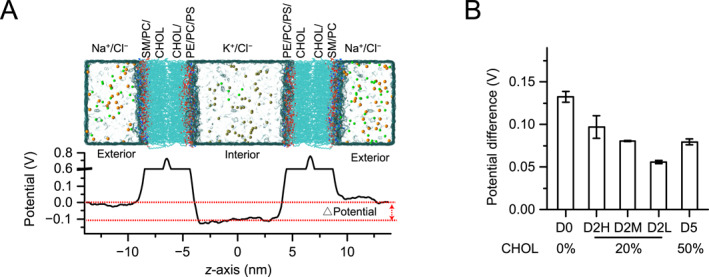
Lipid composition and ion concentration modulate electrostatic potentials across the membrane. (A) Schematic of the PHYSIOL model construction (top). Each PHYSIOL model is built from two lipid bilayer snapshots of the same asymPL model simulation. The inner leaflets of the two bilayers are placed against each other. Two separated regions are then generated (exterior and interior) under periodic boundary conditions, where different ionic conditions can be applied. A representative profile of electrostatic potentials is shown (D2H model, bottom). The electrostatic potential difference between the interior and exterior regions is indicated. (B) Electrostatic potential difference across membrane of the PHYSIOL models. Error bars are SEMs of two independent simulations.

The presence of diverse phospholipids leads to localized variations in ion distribution surrounding the membrane, particularly pronounced for those phospholipids with a negative charge in the inner leaflet. This, combined with varying ion concentrations, contributes to maintain the electrostatic potential difference across membrane, which could influence transmembrane signaling. To address the effect of lipid components and ion concentration on electrostatic potential difference, five models (PHYSIOL) are constructed. Each model contains two lipid bilayers with their inner leaflets placed against each other, replicating the structures that form the asymPL simulation (Figure 5A, Figure S4 and Table S1). Under the periodic conditions in MD simulations, the PHYSIOL setup partitions the system into two separated regions (interior and exterior as shown in Figure 5A), enabling us to simulate different ion conditions that mimic the intracellular and extracellular environments. This approach offers a more physiologically relevant perspective compared to other existing models.

The electrostatic potential is constant in both exterior and interior regions but undergoes a rapid transition at the water/lipid interface, exhibiting a sharp peak in the middle of the lipid bilayer (Figure 5A). This potential profile is similar to previous simulations using chemistry at harvard macromolecular mechanics force field [50, 51], but is slightly different from those using the united‐atom GROMACS force field [26, 32].

The averaged electrostatic potentials were calculated for the exterior (|*z*| > 11 nm) and interior (|*z*| < 3 nm) regions for the PHYSIOL models (Figure 5A). The electrostatic potential in the exterior region surpasses that in the interior region and depends on the ion concentration employed. The electrostatic potential differences are about 92 mV, 67 mV, and 58 mV for PHYSIOL D2H, D2M, and D2L models, all of which share an identical lipid composition (Figure 5B).

Unexpectedly, electrostatic potential differences are also observed in PHYSIOL D0, D2M, and D5 models, despite their similar ion concentrations, with potential differences of about 130 mV, 81 mV, and 78 mV, respectively (Figure 5B, Figure S4). This suggests that lipid composition also contributes to the electrostatic potential difference. An increase in CHOL concentration results in a decrease in electrostatic potential differences (Figure 5B). It is important to note here that increasing CHOL concentration also reduces the 1‐palmitoyl‐2‐oleoyl‐phosphatidylserine (POPS) concentration in the inner leaflet. Negatively charged POPS lipids attract more cations, resulting in a peak of ion concentration at the water/lipid interface in the inner leaflet.

### 2.6 Water permeation across the membrane affected by CHOL concentration and the electrostatic potential difference

Permeation of water molecules across the lipid membrane has been extensively studied, with multiple factors such as membrane thickness, lipid fluidity, and area per lipid being crucial [52, 53, 54]. Previous experimental studies revealed that the permeability of water and metabolic molecules decreases with increasing CHOL concentration in pure dioleoyl‐phosphatidylcholine bilayer [55, 56]. Although rare, water permeation events are indeed observed in our simulations. Consequently, we analyzed the dependence of water permeation on CHOL concentration and electrostatic potential differences.

**FIGURE 6:**
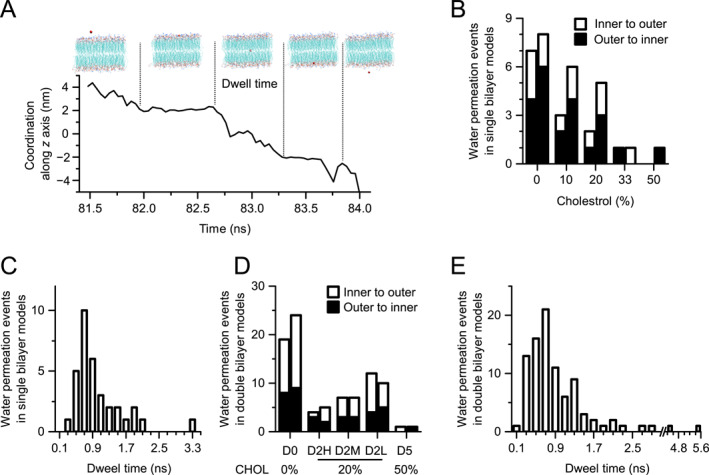
Water permeation across membrane is regulated by cholesterol (CHOL) concentration and electrostatic potential. (A) Snapshots of water permeation across lipid membrane of a representative event (top). The movement of the water along the membrane direction was monitored (bottom), the permeation dwell time is defined as the duration when the water located within the hydrophobic regions of the membrane. (B–C) Number of water permeation events occurred in the asymPL model simulations (B) and their dwell time distributions (C). (D–E) Number of water permeation events occurred in the PHYSIOL model simulations (D) and their dwell time distributions (E). Permeation from outer to inner leaflet or from inner to outer leaflet is indicated with filled or empty bars in (B) and (D). Number of water permeation events in two independent simulations are presented respectively in (B) and (D). The dwell time of water permeation events in two independent simulations is analyzed together in (C) and (E).

A typical event of water permeation is shown in Figure 6A, showcasing a water molecule moving from outer leaflet side to the inner leaflet side. It is worth noting that water molecules could move bidirectionally from inner to outer or vice versa. We counted the number of water permeation events that occurred in the asymPL and PHYSIOL models. For the asymPL model, the results reveal a monotonous decrease in the number of permeation events with increasing CHOL concentration, with a slight predominance of outer‐to‐inner permeation over inner‐to‐outer (Figure 6B). It is worth noting that no permeation was observed for the asymPL H50 model (highest CHOL concentration). The dwell time, defined as the time duration when the water molecule stays within the hydrophobic region, of the total permeation events ranged from about 0.3 to 2.2 ns, with an optimal dwell time of about 0.5–1.0 ns (Figure 6C). Detailed permeation analysis indicates that the water molecule tends to move toward the opposite side of the membrane directly, with no stop events observed.

Analogous to the asymPL models, the PHYSIOL models also exhibit a marked decline in water permeation events as the CHOL concentration increases (Figure 6D). Moreover, in nearly all PHYSIOL models, the number of permeation events from the interior to the exterior exceeds those from the exterior to the interior. For the three PHYSIOL D2 models, the permeation events from the exterior to the interior are almost similar, but the permeation events from the interior to the exterior increase with decreasing ion concentration (Figure 6D). These results suggest that ion concentration and electrostatic potential difference significantly impact the free permeation of water. The distribution of the dwell time of water permeation events in PHYSIOL models is similar to that in asymPL models (Figure 6E). Ion permeation was not observed in any of the simulations.

## 3 DISCUSSION

The asymmetry of phospholipids between the two leaflets is an inherent property of most biological membranes. The asymmetry of CHOL between the leaflets is still under debate, but accumulating evidences suggest its occurrence. Maintaining lipid asymmetry is an energy‐consuming process involving many membrane proteins, including flippases, floppases, and scramblases [57]. Factors such as the fluidity and assemble preference of different lipid molecules, membrane bending by cytoskeleton remodeling, and membrane‐binding proteins may also redistribute lipids locally. These factors can affect membrane properties and, consequently the function of membrane receptor or transporter proteins. Therefore, deciphering the properties of lipid membranes composed of different lipid combinations is of great importance. In this study, we focused on exploring the influence of the diversity of phospholipid head groups alongside CHOL concentration.

CHOL, an important component of the plasma membrane, exhibits ordering and condensing effects, contributing to the formation of lipid‐order regions such as lipid rafts. We studied the effect of CHOL concentration and asymmetry on asymmetric multi‐component lipid bilayers, analyzing mesoscopic properties including lateral pressure, electrostatic potential, and water permeation. Our findings reveal that CHOL not only affects basic membrane properties such as surface area, order parameter, and lipid tilting angle but also has distinct effects on lateral pressure, electrostatic potential, and water permeation.

The differences in pressure and electrostatic potential across the two leaflets of the membrane could regulate the functions of some transmembrane signaling proteins. Additionally, the angle formed between transmembrane helix and membrane plane is related to the activation of specific membrane proteins, such as integrins [58]. Moreover, transmembrane helices tend to exhibit greater tilt angles in thinner lipid bilayer [59]. Our results indicate that the differences of lateral area, thickness, pressure, and electrostatic potential of membranes with different lipid compositions are strongly related to CHOL concentration.

Previous experiments have proven that CHOL preferentially associates with lipids containing two saturated tails to separate the lipid‐ordered domains from disordered domains [60, 61]. Therefore, SM and CHOL are thought to be the primary components of lipid raft [62], indicating high enrichment of CHOL within these domains. Based on our simulation results, the pressure and electrostatic potential at the raft or nonraft region should differ. The membrane thickness decreases significantly for the 0% and 50% CHOL models in our study. This reduction in membrane thickness may influence the angle between transmembrane helices and the membrane plane, potentially altering the function of membrane proteins or receptors.

Permeation of specific small molecules across the membranes is important for cellular communication with surroundings. Our results confirm that the ability of water permeation across the membrane is weakened by CHOL, which is consistent with previous studies [55, 56]. From the results, we could speculate that CHOL may influence the permeation of other chemicals across membrane, including some molecular drugs. Consequently, CHOL‐regulating drugs may be employed in combination with disease‐treating drugs to enhance its efficiency.

MD simulations have been extensively used to investigate the structure–function relationship of membrane proteins. Most of these simulations overlook the intricate nature of membranes by employing simplistic models comprising solely a single lipid component, such as 1‐palmitoyl‐2‐oleoyl‐phosphorylcholine or DPPC. Conversely, the membrane models developed in our study offer a more physiologically relevant alternative for simulating membrane proteins/receptors, thereby enhancing the accuracy and relevance of the simulations.

**Limitations of the study:** The length and saturation of fatty acid of phospholipids are diverse, which are important for membrane function. In this study, the tails of PE, PC, and PS share a common structure comprising one saturated 16‐carbon chain and one unsaturated 18‐carbon chain with one double bond. The tail of SM consists of two saturated 16‐carbon chains. Secondly, low abundance lipids, such as PI, PA, diacylglycerol, and CHOL derivatives, are not considered. Thirdly, the models utilized in this study are very small and near planar lipid bilayer containing only about 120 lipids. That is, although some important features are considered, these membrane models are still far from real cell membrane. To gain a comprehensive understanding of membrane structure and function, it is imperative to employ a larger bilayer model that encompasses a wider array of lipids, including those with varying fatty acid lengths and saturation levels.

## AUTHOR CONTRIBUTIONS

**Yong Zhang**: Investigation; writing – original draft. **Jizhong Lou**: Conceptualization; funding acquisition; supervision; writing – review & editing.

## CONFLICT OF INTEREST STATEMENT

The authors Yong Zhang and Jizhong Lou declare that they have no conflicts of interest or financial conflicts to disclose.

## ETHICS STATEMENT

This article does not contain any studies with human or animal subjects performed by any of the authors.

## Supporting information

Supplementary material

## Data Availability

All data are available in the main text or the supplementary materials.
