# Supplementary material for: Impact of lipid asymmetry on membrane biophysical properties: Insights from molecular dynamics simulations
Source: Quant Biol. 2025 Jan 27;13(2):e89. doi: 10.1002/qub2.89 (PMC12806134; doi:10.1002/qub2.89)
Supplement: Supplementary file 1 — Supplementary material [file QUB2-13-e89-s001.doc]

### Impact of Lipid Asymmetry on Membrane Biophysical Properties: Insights from Molecular Dynamics Simulations

Yong Zhang, and Jizhong Lou*

Key Laboratory of Epigenetic Regulation and Intervention, Institute of Biophysics, Chinese Academy of Sciences, Beijing 100101, China

University of Chinese Academy of Sciences, Beijing 100049, China

**Systems and Methods**

**System setup**

To construct asymmetric membrane models, we initially built two distinct sets of symmetric lipid bilayers, each based on the suggested composition of the outer and inner leaflets of plasma membranes, respectively. Within each set, we established five models, varying the cholesterol (CHOL) concentration incrementally: 0%, 10%, 20%, 33% and 50%. Both leaflets in each of the symmetric models contained 120 lipid molecules, including phospholipids and CHOL. Specifically, the outer leaflet mimicking models (OUTER) featured a 1:1 ratio of 1-palmitoyl-2- -oleoyl-sn-glycero-3-phosphocholine (POPC) and N-palmitoyl sphingomyelin (PSM), while the inner leaflet mimicking models (INNER) consisted of 1-palmitoyl-2-oleoyl- -sn-glycero-3-phosphoethanolamine (POPE), 1-palmitoyl-2-oleoyl-sn-glycero-3- -phosphoserine (POPS), and POPC with a ratio of 2:1:1. The initial coordinates of these models was generated by Membrane Builder plugin in CHARMM-GUI webserver [1]. The xy-plane is designated as the plane of lipid bilayer and the *z*-axis is oriented perpendicularly to the lipid bilayer plane.

The symmetric INNER and OUTER membrane models were subjected to molecular dynamics (MD) simulations to obtain equilibrium membrane structures. Subsequently, the lateral area of each equilibrated model was calculated.

The third set of models (asymPL), with asymmetric lipid composition, was then built after evaluating lateral area of the symmetric models and calculating the required number of lipid molecules to offset the area difference between the INNER and OUTER models, ensuring the asymPL models are stress-balanced between the two leaflets.

The fourth set of models (STRESS) were modified from the asymPL model with 20% CHOL. From this model, we adjusted the lipid count in the outer leaflet by -10%, -5%, +5% to create three asymmetric lipid bilayer models with unmatched lateral areas, generating stress between two leaflets.

The fifth set of models (asymCHOL) were built with different CHOL ratios in both leaflets. This set comprises three models, where the outer leaflets was taken from the OUTER model with 33% CHOL and the inner leaflet was taken from the INNER model with 0%, 10% and 20% CHOL, exhibiting asymmetry for both phospholipids and CHOL.

The sixth set of models (PHYSIOL) were built to achieve differing extracellular and intracellular environments. The model contains two asymmetric lipid bilayers, where the inner leaflet mimic layers were positioned in opposition to each other, maintaining a specific distance. Under the periodic boundary condition employed in the MD simulations, the system contains separated extracellular and intracellular sides, allowing for the application of different ion conditions on either side (see Fig. 4A). The extracellular side was charge-balanced with Na^+^ and Cl^-^, whereas the intracellular side was charge-balanced with K^+^ and Cl^-^. The asymPL models with 0%, 20% and 50% CHOL were used to construct the PHYSIOL models. Moreover, three different ion concentrations shown in Fig. 4A were considered for the PHYSIOL model with 20% CHOL ratio.

Except the PHYSIOL models, 150 mM NaCl was used to neutralize and maintain ion concentration of each model. For the PHYSIOL models, the ion concentrations were shown in Fig. S3.

The detailed information of all models built and simulated in this study is listed in Table S1.

**MD Simulations**

All systems are energy minimized using conjugate gradient method in three steps: 1) 5000 steps energy minimization with all water molecule constrained; 2) 5000 steps energy minimization with P atoms of phospholipids and O3 atoms of CHOL constrained; 3) 5000 steps energy minimization without any constrain. Afterwards, three-step MD simulations in NVT ensemble are performed: 1) 1 ns MD simulation with P atoms, O3 atom, and all water molecules constrained; 2) 2 ns MD simulation with all water molecules constrained; 3) 2 ns MD simulation without any constrain. Finally, production MD simulations are carried out to equilibrate the system in the NPT ensemble. The simulation time for each model is listed in Table S1. The total simulation time is ~6.7 microseconds.

In MD simulations for asymmetric lipid bilayers, we maintain a consistent time scale of approximately 200 ns for each model to quantify their biophysical properties. Conversely, for a symmetric lipid bilayer, our primary goal is to equilibrate its lateral area to quantify differences in molecular count between the inner and outer leaflets, facilitating asymmetric membrane assembly. Hence, once the system's lateral area achieves equilibrium and we have captured the essential data, the simulation will be terminated.

During simulations, Particle Mesh Ewald (PME) method is used to reduce the truncation error of electrostatic interactions, and the grid spacing is set to 0.1 nm. Van der Waals (VDW) interactions are computed using the switch method, in which the Lennard-Jones (LJ) 6-12 potential is calculated normally within 1.0 nm, and switches off smoothly between 1.0 to 1.2 nm. The SHAKE method is used on all hydrogen-containing bonds to allow a 2 fs time step in the equilibration simulations. Temperature (310 K) is controlled using Langevin dynamics method and the damping coefficient is set to 1/ps. Pressure (1 atm) is controlled using the Langevin piston Nose-Hoover method in a semi-isotropic manner, so no external stress was applied to both layers. All energy minimizations and MD simulations are carried out using the NAMD2.8 software package. CHARMM36 force field was used in all simulations. Force field parameters for PSM are from reference [2]. All system preparations and illustrations were performed using VMD [3].

**Analysis**

Analysis of the MD trajectories was performed using GROMACS software package [4] and the VMD program with a set of self-coded scripts. The surface area of lipid bilayer is defined as the area of xy-plane of the periodic cell. The thickness of the lipid bilayer was defined as the distance between the maximal mass densities of lipids in each leaflet. The order parameters were calculated and averaged by the atoms ranging from C2 to terminal C atom of the fatty acid chains. The tilting angle of phospholipids was defined as the angle formed by the vector connecting the center of mass (COM) of phospholipid head region and the tail region and the xy-plane of the simulation water box. The tilt angle of CHOL is defined as the angle formed by the vector connecting the COM of first and third hexatomic ring and the xy-plane of the simulation water box. The electrostatic potentials across the PHYSIOL models was computed and averaged by evaluating the double integral of the charge density, and the detail calculation method can be found in GROMACS user manual [5].

The lateral pressure profile as a function of z coordinate was defined as the difference between the lateral component [*P*_L_ = (*P*xx+*P*yy)/2] and the normal components [*P*_N_ = *P*zz], where *P*xx, *P*yy, and *P*zz denote the diagonal elements of the pressure tensor. Every diagonal element of the tensor, comprised of four terms: kinetic energy, bonded interactions, nonbonded interactions, and an Ewald sum, are calculated using NAMD 2.8, and the algorithm is adopted from Lindahl and Edholm [6], with modifications to enable Ewald sums from Sonne [7]. The profiles were calculated with 120 slabs for the PHYSIOL models, corresponding to an approximate slab width of 0.1 nm, which has been proven to be reasonable to evaluate pressure profiles. The contribution to the pressure from non-bonded interactions was computed using a cutoff of 1.8 nm [8], and a smoothing function (B-spline curve) is applied to all computed pressure profiles.

Table S1 Lipids composition of lipid bilayer models simulated in this study

|  | Name | CHOL% | Outer leaflet / Inner leaflet | Simulation Time |
| --- | --- | --- | --- | --- |
| OUTER | O00 | Zero | 60 POPC + 60 PSM / * | ~140ns |
|  | O10 | 10% | 54 POPC + 54 PSM + 12 CHOL / * | ~160ns |
|  | O20 | 20% | 48 POPC + 48 PSM + 24 CHOL / * | ~160ns |
|  | O33 | 33% | 40 POPC + 40 PSM + 40 CHOL / * | ~140ns |
|  | O55 | 50% | 30 POPC + 30 PSM + 60 CHOL / * | ~125ns |
|  |  |  |  |  |
| INNER | I00 | Zero | 60 POPE + 30 POPC + 30 POPS / * | ~120 ns |
|  | I10 | 10% | 54 POPE + 27 POPS + 27 POPC + 12 CHOL / * | ~185ns |
|  | I20 | 20% | 48 POPE + 24 POPS + 24 POPC + 24 CHOL / * | ~160ns |
|  | I33 | 33% | 40 POPE + 20 POPS + 20 POPC + 40 CHOL / * | ~130ns |
|  | I55 | 50% | 30 POPE + 15 POPS + 15 POPC + 60 CHOL / * | ~110ns |
|  |  |  |  |  |
| asymPL | H00 | Zero | 60 POPC + 60 PSM /  62 POPE + 32 POPS + 31POPC | ~200ns * 2 |
|  | H10 | 10% | 54 POPC + 54 PSM + 12 CHOL /  55 POPE + 28 POPS + 27 POPC + 12 CHOL | ~200ns * 2 |
|  | H20 | 20% | 50 POPC + 50 PSM + 26 CHOL /  48 POPE + 24 POPS + 24 POPC + 24 CHOL | ~200ns * 2 |
|  | H33 | 33% | 42 POPC + 42 PSM + 41 CHOL /  40 POPE + 20 POPS + 20 POPC + 40 CHOL | ~200ns * 2 |
|  | H50 | 50% | 31 POPC + 31 PSM + 60 CHOL /  30 POPE + 15 POPS + 15 POPC + 60 CHOL | ~200ns * 2 |
|  |  |  |  |  |
| STRESS | +5% | 20% | 52 POPC + 52 PSM + 28 CHOL /  48 POPE + 24 POPS + 24 POPC + 24 CHOL | ~210ns |
|  | -5% | 20% | 48 POPC + 48 PSM + 24 CHOL /  48 POPE + 24 POPS + 24 POPC + 24 CHOL | ~210ns |
|  | -10% | 20% | 46 POPC + 46 PSM + 22 CHOL /  48 POPE + 24 POPS + 24 POPC + 24 CHOL | ~210ns |
|  |  |  |  |  |
| asymCHOL | 0% | 33% / 0% | 52 POPC + 52 PSM + 52 CHOL /  60 POPE + 30 POPS + 30 POPC | ~210ns |
|  | 10% | 33% / 10% | 52 POPC + 52 PSM + 52 CHOL /  54 POPE + 27 POPS + 27 POPC + 12 CHOL | ~210ns |
|  | 20% | 33% / 20% | 52 POPC + 52 PSM + 52 CHOL /  48 POPE + 24 POPS + 24 POPC + 24 CHOL | ~210ns |
|  |  |  |  |  |
| PHYSIOL | D0 | Zero | 60 POPC + 60 PSM /  62 POPE + 32 POPS + 31POPC | ~210ns * 2 |
|  | D2H | 20% | 50 POPC + 50 PSM + 26 CHOL /  48 POPE + 24 POPS + 24 POPC + 24 CHOL | ~210ns * 2 |
|  | D2M | 20% | Same with D2H | ~200ns * 2 |
|  | D2L | 20% | Same with D2H | ~200ns * 2 |
|  | D5 | 50% | 31 POPC + 31 PSM + 60 CHOL /  30 POPE + 15 POPS + 15 POPC + 60 CHOL | ~200ns * 2 |

*: The components of outer and inner leaflet are same for OUTER and INNER models.


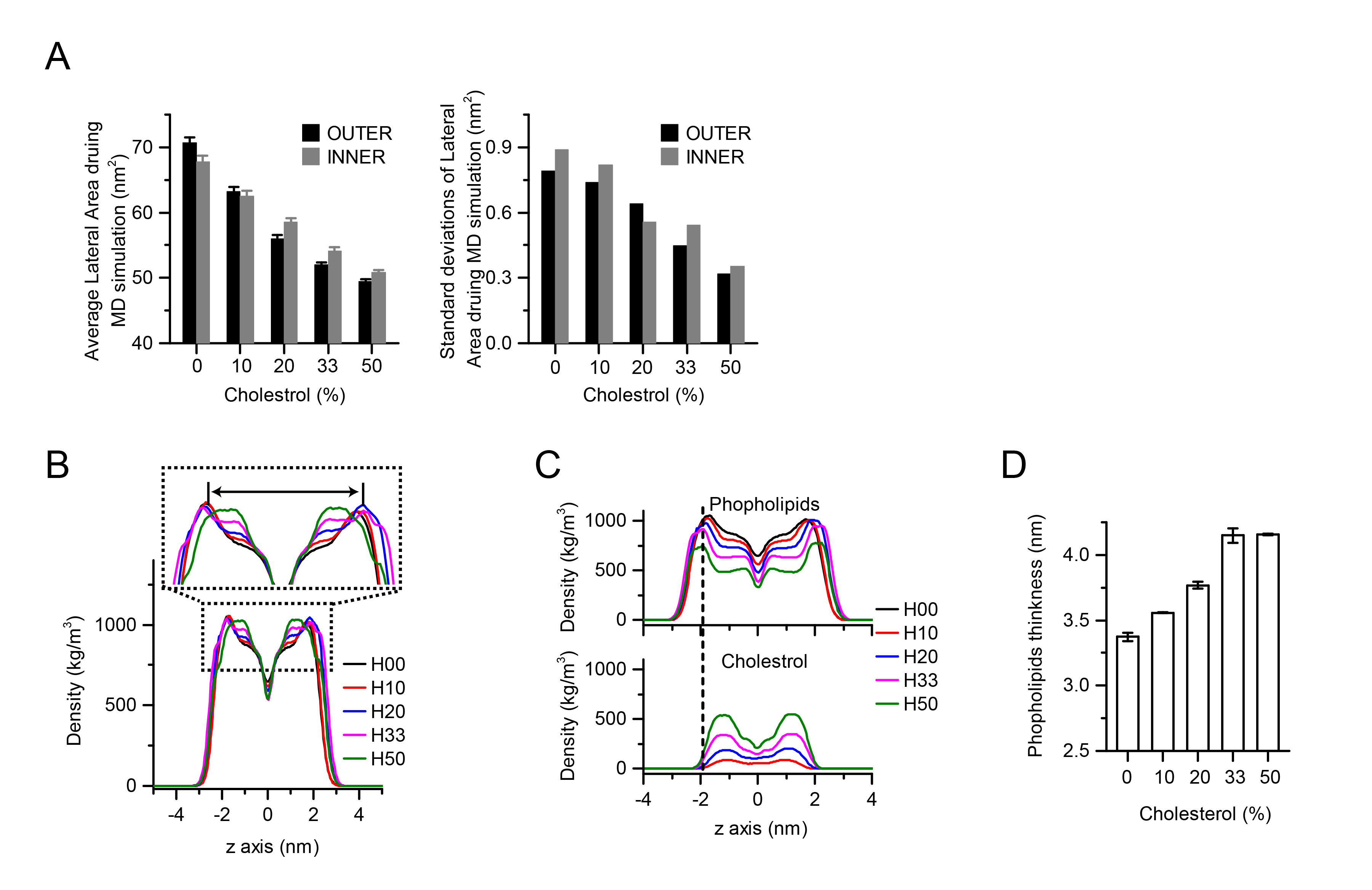


**Figure S1. Properties of the asymPL models.**

A) Average lateral areas and their standard deviations of five asymPL models during the simulations.

B) Total mass density distribution of phospholipids and CHOL along the normal direction of the lipid bilayer in the asymPL models. The density distribution around the lipid head region is highlighted. The thickness of the membrane is defined as the distance between two maximum values of the density distribution for each model.

C) Mass density distribution of phospholipids and CHOL along the normal direction of the lipid bilayer in the asymPL models, respectively.

D) The phospholipid’s thickness in the asymPL models.


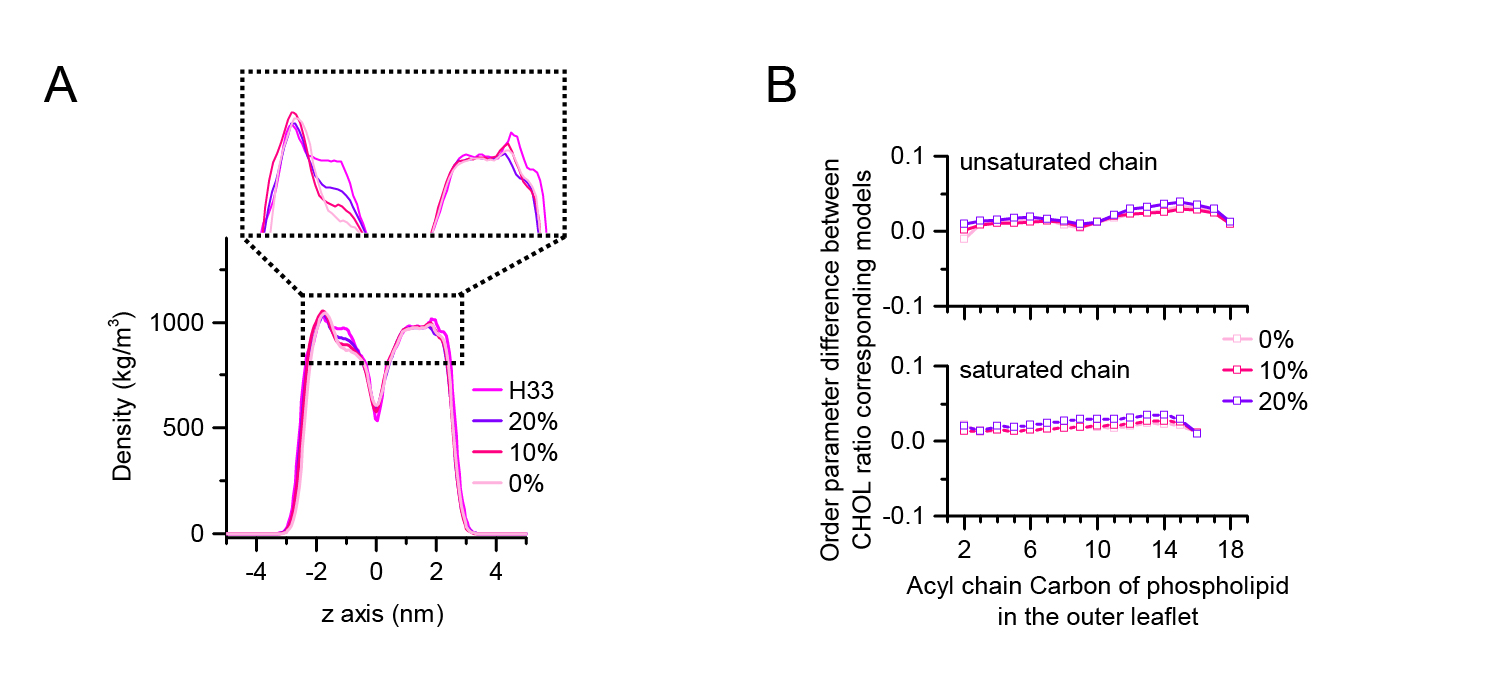


**Figure S2, Density distribution and order parameter difference of the asymCHOL models**.

A) Density distribution along the normal direction of the lipid bilayer for the asymCHOL models compared with that for asymPL H33 model. The density distribution around lipid head regions is highlighted.

B) The order parameter difference between the asymCHOL models and the asymPL H33 model for unsaturated (top) and saturated (bottom) lipid tail atoms in the outer leaflet.


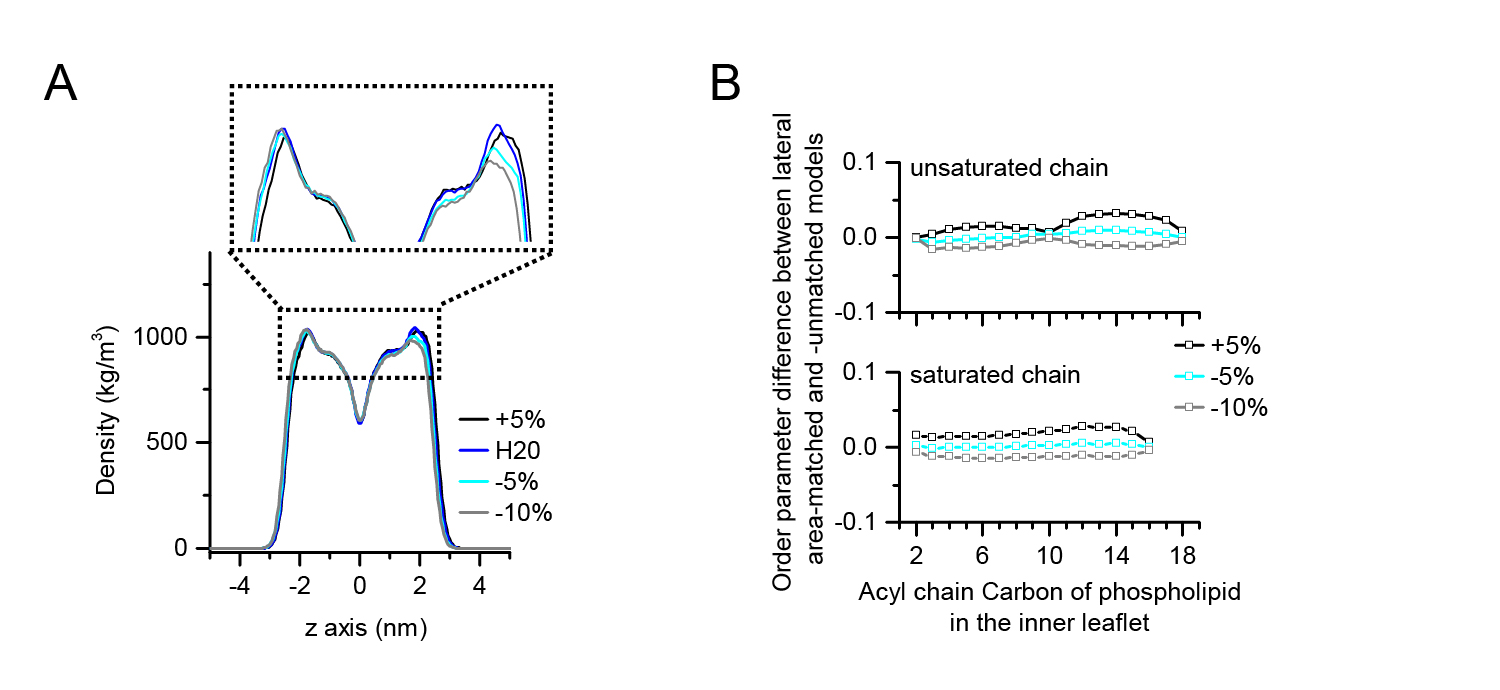


**Figure S3, Density distribution and order parameter difference of the STRESS models.**

A) Density distribution along the normal direction of the lipid bilayer of the STRESS models compared with that of asymPL H20 model. The density distribution around lipid head region is highlighted.

B) Order parameter difference between the STRESS models and the asymPL H20 model for unsaturated (top) and saturated (down) lipid tail atoms in the inner leaflet.


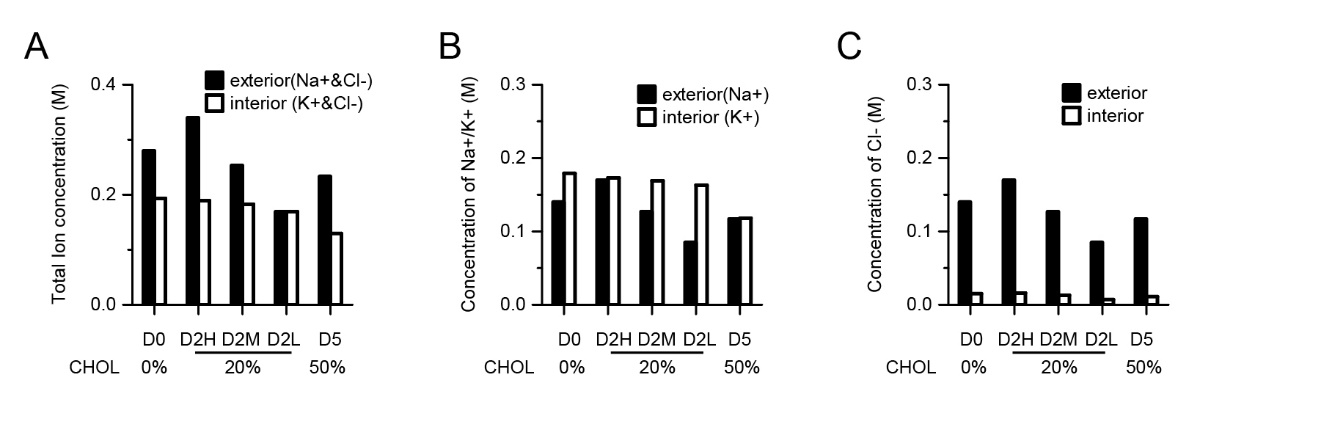


**Figure S4, Ion concentration in the PHYSIOL models.**

A) Ion species and their total concentration in the exterior and interior regions used in the PHYSIOL models. The number of different ions was calculated to maintain the desired concentration in each region and also neutralize the charge in the region.

B) Concentration of the Na+ or K+ ions in the exterior or interior region of the PHYSIOL models.

C) Concentration of the Cl- ions in the exterior and interior region of PHYSIOL models.

Reference

1. Jo, S., Lim, J. B., Klauda, J. B., and Im, W. (2009) Charmm-gui membrane builder for mixed bilayers and its application to yeast membranes. Biophys J. 97, 50-58

2. Hyvönen, M. T., and Kovanen, P. T. (2003) Molecular dynamics simulation of sphingomyelin bilayer. J Phys Chem B. 107, 9102-9108

3. Humphrey, W., Dalke, A., and Schulten, K. (1996) Vmd: Visual molecular dynamics. J Mol Graph 14, 33-38

4. Hess, B., Kutzner, C., Spoel, D. v. d., and Lindahl, E. (2008) Gromacs 4: Algorithms for highly efficient, load-balanced, and scalable molecular simulation. J Chem Theory Comput. 4, 435-447

5. Spoel, D. v. d., Lindahl, E., Hess, B., Buuren, A. R. v., Apol, E., Meulenhoff, P. J., Tieleman, D. P., Sijbers, A. L. T. M., Feenstra, K. A., Drunen, R. v.*, et al.* (2010) Gromacs user manual version 4.5.4.

6. Lindahl, E., and Edholm, O. (2000) Spatial and energetic-entropic decomposition of surface tension in lipid bilayers from molecular dynamics simulations J Chem Phys 113, 3382-3393

7. Sonne, J., Hansen, F. Y., and Peters, G. H. (2005) Methodological problems in pressure profile calculations for lipid bilayers J Chem Phys. 122, 124903-124911

8. Gullingsrud, J., and Schulten, K. (2004) Lipid bilayer pressure profiles and mechanosensitive channel gating. Biophys J. 86, 3496-3509
